# The Association Between the Atherogenic Index of Plasma and Cognitive Function: Evidence From the NHANES 2011–2014

**DOI:** 10.1002/brb3.70768

**Published:** 2025-08-22

**Authors:** Pingping Huang, Gaocan Ren, Yifei Wang, Yicheng Liu, Hongwei Zhang, Shuangqing Fu, Zhibo Zhang, Lijun Guo, Xiaochang Ma

**Affiliations:** ^1^ Xiyuan Hospital China Academy of Chinese Medical Sciences Beijing China; ^2^ Graduate School China Academy of Chinese Medical Sciences Beijing China; ^3^ Xiyuan Hospital Beijing University of Chinese Medicine Beijing China; ^4^ National Clinical Research Center for Chinese Medicine Cardiology Beijing China

**Keywords:** atherogenic index of plasma, cognitive function, cross‐sectional study, NHANES

## Abstract

**Background:**

Atherosclerosis is the primary cause of cerebrovascular disease, which in turn has substantial deleterious impacts on cognitive abilities. The atherogenic index of plasma (AIP), calculated as the logarithmic transformation of the ratio of triglycerides to high‐density lipoprotein cholesterol (log[TG/HDL‐C]), has emerged as a novel biomarker reflecting the balance between proatherogenic and antiatherogenic lipoproteins. Nevertheless, the association between AIP and cognitive function has not been examined in a large cohort. This study examined the relationship between AIP and cognitive performance in a nationally representative cohort of adult Americans.

**Methods:**

A cross‐sectional study was conducted using data from the 2011–2014 National Health and Nutrition Examination Survey (NHANES). The relationship between AIP and cognitive function was examined using several multivariate regression models with adjustment for arrays of potential confound factors. Fitted smoothed curves and threshold effect analysis were used to characterize possible nonlinear relationships.

**Results:**

The study population comprised 995 adults (mean age 63.01 ± 14.93 years; 51.86% female), with 49.65% identifying as non‐Hispanic White, 9.15% as Mexican American, and 20.2% as non‐Hispanic Black. Higher AIP was associated with greater risk of cognitive impairment. Negative correlations were detected between the AIP and both Animal Fluency Test (AFT) performance (β = ‐5.54, 95%CI: ‐9.26, ‐1.82, *p* < 0.05) and Digit Symbol Substitution Test (DSST) performance (β = ‐15.79, 95% CI: ‐26.32, ‐5.27, *p* < 0.05) after adjusting for multiple confounding variables.

**Conclusion:**

Elevated AIP is associated with cognitive impairment in adult Americans.

## Graphical abstract

AIP may serve as a **novel biomarker** for identifying individuals at risk of cognitive decline. Early lipid management could mitigate cerebrovascular damage and preserve executive function.

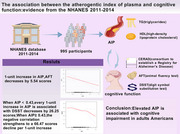

## 1 Introduction

The number of adults with dementia is rising rapidly due to population aging and gains in longevity achieved over previous decades (Avan and Hachinski 2023). Lowering the prevalence of cognitive impairment and the rate at which mild cognitive impairment progresses to dementia are two of the main objectives of geriatric neurology. Current pharmacological interventions for cognitive impairment, such as cholinesterase inhibitors (donepezil, rivastigmine) and N‐Methyl‐D‐Aspartate receptor antagonists (memantine), are primarily used to symptomatically manage cognitive decline in progressive neurological diseases, though their ability to modify underlying neurodegenerative processes remains limited. (Reuben et al. 2024). Despite these developments, the prevalence of dementia continues to increase, with no decrease in global burden expected over the coming decades (Reuben et al. 2024). An early focus on mitigating risk factors may prevent dementia (Livingston et al. 2020), greatly improve quality of life among the aged population, and reduce both caregiver burden and medical costs.

The atherogenic index of plasma (AIP) is a composite indicator of metabolic status proposed by Dobiásová that combines triglyceride (TG) and high‐density lipoprotein cholesterol (HDL) levels (Dobiásová 2004). Initially, AIP was used to assess the risk of cardiovascular disease, and it has been confirmed to predict disease status with reasonable accuracy. For instance, receiver operating characteristic (ROC) curve analysis for predicting coronary artery disease (CAD) yielded an area under the curve of 0.733 and a Youden index of 0.382 (L. Wang et al. 2021). The AIP has now been measured in a variety of populations and disease states, and a cross‐sectional study involving 10,099 adults revealed a significant positive correlation between AIP and both prediabetes and diabetes (for each unit increase in AIP, the odds ratio was found to be 2.49, with a 95% confidence interval of 1.75 to 3.54, *P*< 0.05) (Shi and Wen 2023). Recent studies research has identified a connection between AIP and changes in brain structure, including white matter hyperintensity (WMH), which in turn is a strong risk factor for dementia (Kwon et al. 2022), further suggesting that AIP may be predictive of future cognitive dysfunction. In addition, there is evidence to suggest that there is a strong correlation between dyslipidemia and cognitive impairment (Zhao et al. 2024). Therefore, the association between the AIP index, which includes blood lipid indicators, and cognitive function warrants further exploration. Based on the above background, this study used National Health and Nutrition Examination Survey (NHANES) data to investigate the relationship between AIP and cognitive function.

## 2 Methods

### 2.1 Rationale for Data Selection

The 2011–2014 NHANES cycles were selected for three reasons: (1) these years provide the most recent publicly available data with standardized cognitive assessments, (2) the lipid measurement protocols (enzymatic assays for TC/HDL) and cognitive testing methodologies during this period are methodologically consistent and widely recognized, (3) this timeframe aligns with critical updates in dyslipidemia management guidelines, enhancing the clinical relevance of our findings.

### 2.2 Data Sources and Participants

**FIGURE 1:**
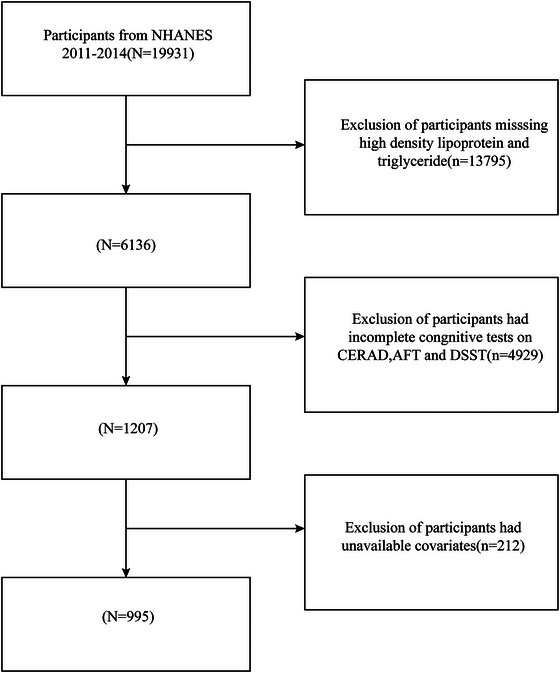
The participant selection flowchart.

The National Center for Health Statistics (NCHS) provided NHANES data from 2011 to 2014 for the current cross‐sectional investigation. Sociodemographic data, such as age, sex, and educational attainment, were gathered through health interviews. Participants provided written informed consent, and the NCHS Research Ethics Committee authorized all procedures (http://www.cdc.gov/nchs/nhanes.htm). Participants with incomplete AIP and covariate data as well as those with incomplete cognitive information were not included in this study. The final sample included 995 participants, and the specific screening process is shown in Figure 1.

### 2.3 AIP

The Centers for Disease Control and Prevention (CDC) provided a standard methodology for the collection and measurement of blood samples for the NHAENS. Precipitation or direct immunoassay was used to detect serum HDL (Bucholz et al. 2018). Additionally, serum TC and HDL values were determined enzymatically (Doran et al. 2015) using a Hitachi 704 Analyzer (Boehringer Mannheim Diagnostics, Indianapolis, IN, USA). Additional details on laboratory testing are available on the CDC website. Formulae to calculate AIP were derived from log [TG (mmol/L)/HDL (mmol/L)]. For improved accuracy and detection of nonlinear associations, we divided AIP into quartiles (Q1: −0.93 to −0.28, Q2: −0.28 to −0.07, Q3: −0.07 to 0.13, Q4: 0.13 to 1.15).

### 2.4 Cognitive Function

Participants in the NHANES completed a battery of cognitive function tests. The Consortium to Establish a Registry for Alzheimer's Disease—Immediate Recall and Delayed Recall Tests (CERAD‐IRT and CERAD‐DRT) were used to evaluate language acquisition skills and short‐term memory. For the IRT, participants were read ten unrelated words aloud and asked to quickly recall as many as they could. In the DRT, the test was conducted 8 to 10 min after presentation of the words (Girbardt et al. 2021). Participants were scored between 0 and 10 according to the number of words recalled. The Animal Fluency Test (AFT) required participants to rapidly generate a list of distinct animal names within one minute, providing an assessment of both verbal and executive cognitive functions. For every animal that was named, one point was given (Clark et al. 2009). Participants also performed the digit symbol substitution test (DSST), a timed evaluation of executive function and processing speed in which participants are requested to transcribe symbols to numbers according to a legend. Participants had two minutes to match symbols to numbers in the 133 boxes and received one point per match (yielding a score from 0 to 133) (Salthouse 1992). Based on previously published research, the 25th percentile of cognitive test scores for each assessment was established as the cutoff (Dong et al. 2020), with participants scoring below the cutoff value defined as having low cognitive function.

### 2.5 Adjustable Covariates

The covariates in this study included the continuous variables age, body mass index (BMI, kg/m^2^), TG (mmol/L), and HDL (mmol/L), and categorical variables included sex, race, education level, marital status, smoking status, alcohol consumption, and presence/absence of hypertension, heart failure, coronary heart disease, stroke, and diabetes. Demographic information such as age, sex (male or female), poverty income ratio (PIR), race (non‐Hispanic white, non‐Hispanic black, Mexican American, other Hispanic American, or other), smoking status (lifetime smokers defined as ≥ 100 cigarettes), and alcohol consumption (consumption of ≥ 12 alcoholic beverages per year defined as alcohol users) was collected from interviews. Body weight was measured using a medical scale with participants dressed in light clothing and without shoes. Body mass index was calculated as weight in kg divided by the square of height in meters (kg/m2). It is worth noting that although AIP is derived from HDL and TG (log(TG/HDL)), these variables were included as covariates in Model 2 to disentangle their independent effects from the composite AIP metric. This approach ensures that associations between AIP and outcomes are not confounded by residual relationships between HDL/TG and other risk factors (e.g., diabetes, obesity).

### 2.6 Statistical Analysis

All statistical analyses were conducted using R, version 4.2.0 (R Foundation) and EmpowerStats (http://www.empowerstats.com, X&Y Solutions, Inc., Boston, MA), and a *p* < 0.05 was considered statistically significant for all tests. Continuous variables are expressed as mean ± standard deviation (SD) if normally distributed or median (interquartile range). All categorical variables are expressed as proportions. Continuous variables were compared by independent sample. Student's t‐tests if normally distributed or the Mann–Whitney U‐test if non‐normally distributed, while categorical variables were compared by chi‐square test. Regression models were established to evaluate the relationship between AIP and cognitive status. In Model 1, data were adjusted for age and sex. In model 2, results were adjusted for sex, age, race, education, marital status, PIR, hypertension, heart failure, coronary heart disease, stroke, smoking, diabetes, drink, TG, HDL, and BMI. For examining the relationship between AIP and low cognitive function, cognitive function was divided into three modules, CERAD, AFT, and DSST. In the analysis of each module, the case group (low cognitive function) was defined as participants with scores below the cut‐off value, while the control group consisted of participants with scores above the cut‐off value. After adjusting for the relevant variables in Models 1 and 2, multivariate logistic regression was used to explore the relationship between AIP and low cognitive function. In this study, participants with missing data for the primary exposure or outcome variables were directly excluded to ensure the rigor of the analytical framework. For covariates, a stratified handling approach was employed: covariates with missing data below 20% were imputed using appropriate statistical methods, while covariates with missing data exceeding 20% were excluded from the final multivariable regression model to avoid potential bias arising from excessive imputation. We took the complex sampling design of NHANES into account in all statistical analyses. Specifically, following the recommendations of the National Center for Health Statistics (NCHS), we used appropriate sample weights, stratification variables, and primary sampling units (PSUs) to ensure that the results are nationally representative and provide unbiased variance estimates. (https://wwwn.cdc.gov/nchs/nhanes/analyticguidelines.aspx#estimation‐and‐weighting‐procedures).

This study also employed a Generalized Additive Model (GAM) to analyze the nonlinear relationships between AIP and cognitive performance. The analysis utilized the Smooth Curve Fitting (SCF) method to illustrate the impact of AIP on cognitive performance. If the resulting effect graph indicated a nonlinear trend, such as curvature or fluctuations, it supported the hypothesis of a nonlinear relationship. Leveraging the detection of nonlinearity, a recursive algorithm was employed to pinpoint significant inflection points between AIP and cognitive performance, facilitating threshold analysis. Results for dichotomous variables are expressed as the odds ratio (OR) and continuous variables as β. The interaction analysis included stratification analysis and interaction tests in the regression model to assess the effect of different subgroup variables on AIP and cognitive function.

### 2.7 Subgroups Analysis

To explore potential effect modifiers and improve clinical interpretability, we conducted subgroup analyses based on biological plausibility and clinical relevance to the association between AIP and cognitive function. (1) Gender. Sex hormones (e.g., estrogen) modulate lipid metabolism and cerebrovascular function, potentially influencing AIP‐cognition associations (Ansere et al. 2025). (2) Age: Older adults are at higher risk for cognitive decline, and AIP's impact may differ by age‐related vascular changes. Advanced age is associated with increased arterial stiffness and microvascular dysfunction, which may amplify AIP's neurotoxic effects (Kleindorfer et al. 2021). (3) BMI: Obesity is linked to chronic inflammation and neurodegeneration, potentially interacting with AIP's atherogenic effects (Asimakidou et al. 2025). Visceral adipose tissue‐derived inflammatory cytokines may mediate this relationship (Elzinga et al. 2023). (4) PRI: Socioeconomic status influences access to healthcare and lifestyle factors mediating cognitive health. Low PRI is associated with poor diet quality and reduced physical activity, exacerbating AIP‐related cerebrovascular risk (Ni et al. 2023). (5)Cardiovascular/Metabolic Comorbidities: Hypertension/Heart Failure/Coronary Heart Disease (CHD)/Stroke/Diabetes: These conditions share pathophysiological pathways with AIP (e.g., insulin resistance, oxidative stress) and may confound or modify its cognitive effects (Einstad et al. 2021; Kang and Malvaso 2023; Matsue et al. 2020; Santisteban et al. 2023; Srikanth et al. 2020).

## 3 Outcomes

### 3.1 Basic Information of Participants

In total, 995 participants from the NHANES (2011–2014) were included in the current study. The mean age of all affected patients was 63.01 ± 14.93 years, and 51.86% were female. The mean scores on the CERAD, AFT, and DSST were 25.35 ± 6.57, 16.63 ± 5.39, and 45.98 ± 16.77, respectively. Compared to participants with low AIP, those with high AIP were more likely to be male, have lower HDL levels, lower CERAD and DSST scores, higher BMI and TG levels, and greater prevalence of drinking, smoking, hypertension, heart failure, diabetes, and near‐diabetic status Table 1.

**TABLE 1:** Baseline characteristics of participants.

Characteristics	Overall (n = 995)	Q1 (‐0.93 to ‐0.28)	Q2 (‐0.28 to ‐0.07)	Q3 (‐0.07 to 0.13)	Q4 (0.13 to 1.15)	*p* value
Age	63.01 ± 14.93	62.79 ± 14.26	60.40 ± 17.24	61.17 ± 16.03	60.89 ± 13.86	0.329
Gender(%)						< 0.001
Male	48.14	39.9	43.01	48.44	60.42	
Female	51.86	60.1	56.99	51.56	39.58	
BMI (%)						< 0.001
< 25	30.45	46.74	37.24	24.29	13.28	
25–29.9	34.77	33.22	35.76	35.39	36.09	
≥ 30	34.77	20.03	27	40.32	50.64	
Race (%)						0.289
Mexican American	9.15	4.24	3.41	4.76	4.93	
Other Hispanic	8.64	2.38	3.57	6.29	3.94	
Non‐Hispanic White	49.65	77.15	77.31	74.65	80.57	
Non‐Hispanic Black	20.2	10.14	10.81	7.69	4.62	
Other Race	12.36	6.08	4.90	6.61	5.94	
Marital status (%)						0.209
Married	56.28	62.27	65.58	59.43	63.26	
Widowed	14.87	14.59	11.63	13.68	10.15	
Divorced	12.06	7.06	8.49	14.31	8.99	
Separated	2.71	2.35	0.33	2.27	1.46	
Never married	9.65	10.60	9.79	8.04	11.04	
Living with partner	4.43	3.14	4.19	2.27	5.11	
Education (%)						0.060
Below high school	22.71	11.87	18.36	12.58	18.19	
high school	23.52	18.95	19.50	25.40	23.76	
Above high school	53.77	69.18	62.13	62.02	58.06	
PRI (%)						0.446
≤ 1	18.39	9.19	13.50	9.90	10.90	
> 1	81.61	90.81	86.50	90.10	89.10	
Drinking (%)						
yes	66.83	67.20	63.96	70.51	75.19	0.042
no	33.17	32.80	36.04	29.49	24.81	
Smoking (%)						< 0.001
yes	52.96	44.77	47.46	61.57	58.20	
no	47.04	55.23	52.54	38.43	41.80	
Hypertension (%)						< 0.001
yes	63.72	47.71	65.20	68.35	63.92	
no	36.28	52.29	34.80	31.65	36.08	
Heart failure (%)						0.018
yes	7.34	5.81	4.77	10.03	11.17	
no	92.66	94.19	95.23	89.97	88.83	
CHD (%)						0.184
yes	9.85	10.91	10.00	8.82	14.61	
no	90.15	89.09	90.00	91.18	85.39	
Stroke (%)						0.293
yes	6.53	8.05	6.13	10.34	6.48	
no	93.47	91.95	93.87	89.66	93.52	
Diabetes (%)						< 0.001
yes	21.91	9.70	16.76	25.02	29.84	
no	72.56	85.84	77.29	70.36	65.02	
borderline	5.53	4.46	5.95	4.61	5.13	
TG	1.36 ± 0.85	0.68 ± 0.18	1.03 ± 0.21	1.38 ± 0.27	2.43 ± 0.99	< 0.001
HDL	1.43 ± 0.43	1.87 ± 0.44	1.54 ± 0.29	1.30 ± 0.22	1.07 ± 0.22	< 0.001
CERAD	25.35 ± 6.57	27.25 ± 6.59	26.07 ± 6.46	25.97 ± 6.81	25.52 ± 6.11	0.018
AFT	16.63 ± 5.39	17.75 ± 5.51	17.07 ± 5.48	17.04 ± 5.47	17.32 ± 5.46	0.451
DSST	45.98 ± 16.77	50.61 ±16.45	50.38 ± 17.58	46.05 ± 16.98	49.36 ± 14.91	0.008

*Note*: Values are presented as mean ± standard deviation (SD) for continuous variables or percentages for categorical variables.

Abbreviations: AFT: animal fluency test; CERAD: consortium to establish a registry for Alzheimer's disease; CHD: coronary heart disease; DSST: digit symbol substitution test; HDL: high density lipoprotein; PRI: Ratio of family income to poverty; TC: total cholesterol; The CERAD test included CERAD‐IRT (immediate recall test) and CERAD‐DRT (delayed recall test). Continuous variables are shown as mean (SD) and categorical variables as percentages.

Table 2 summarizes the associations between AIP and cognitive test scores. Higher AIP was associated with a significantly increased risk of cognitive impairment. After adjusting for all confounding factors, a negative relationship was observed between the AIP and cognition function related to AFT (β = ‐5.54, 95%CI: ‐9.26, ‐1.82, *p* < 0.05) and DSST (β = ‐15.79, 95% CI: ‐26.32, ‐5.27, *p* < 0.05). To assess the robustness of these associations, AIP was further converted into a categorical variable (quartiles). The fourth quartile of the AIP index was significantly associated with AFT score (β = ‐2.57, 95% CI: ‐4.29, ‐0.86, *p* < 0.01) while the third quartile was significantly correlated with DSST score (β = ‐3.75, 95% CI: ‐7.26, ‐0.25, *p* < 0.05). No significant correlations were detected between the AIP index and CERAD test score.

**TABLE 2:** Multivariable linear regression to assess the association of AIP with cognitive function.

CERAD				AFT			DSST		
	Crude	Model 1	Model 2	Crude	Model 1	Model 2	Crude	Model 1	Model 2
AIPindex (continuous)	−2.04(−3.30,−0.78)*	−1.95(−3.23,−0.66)*	−5.35(−9.92,0.78)	0.03 (‐1.04, 1.09)	−0.16(−1.24, 0.92)	−5.54(−9.26, −1.82)*	−2.01 (−5.22, 1.20)	−1.56(−4.83, 1.71)	−15.79 (−26.32, −5.27) *
AIPindex (quartiles)									
Q1	Reference			Reference			Reference		
Q2	−1.18(−2.33, 0.03)	−1.15 (−2.30, 0.00)	−0.92(−2.19, 0.35)	−0.68(−1.65, 0.29)	−0.67(−1.64, 0.30)	−0.83(−1.86, 0.20)	−0.22 (−3.14, 2.69)	−0.22 (−3.14, 2.71)	0.75 (−2.18, 3.67)
Q3	−1.28(−2.43, −0.13)*	−1.24 (−2.39, −0.09) *	−1.02(−2.54, 0.51)	−0.70(−1.67, 0.27)	−0.75(−1.72, 0.22)	−1.34(−2.57, −0.10)*	−4.56(−7.47,−1.65)*	−4.44 (−7.36, −1.52) *	−3.75 (−7.26, −0.25) *
Q4	−1.74(−2.85, −0.62)*	−1.66 (−2.78, −0.53) *	−1.66(−3.76, 0.45)	−0.43(−1.37, 0.51)	−0.57(−1.52, 0.38)	−2.57(−4.29, −0.86)*	−1.25 (−4.07, 1.57)	−0.92 (−3.77, 1.93)	−2.65 (−7.50, 2.19)

Abbreviations: AFT: Animal Fluency test; DSST: digit symbol substitution test. Data are presented as β (95% confidence intervals). * P < 0.05. Crude model adjusted for None; Model 1 adjusted for age and gender; Model 2 adjusted for gender, age, race, education, marital status, PIR, hypertension, heart failure, coronary heart disease, stroke, smoking, diabetes, drink, TG, HDL, BMI; CERAD: The Consortium to Establish a Registry for Alzheimer's Disease.

### 3.2 Subgroup Analysis

Subgroup analyses and interaction tests were performed to verify the stability of the associations between AIP and cognitive function (Supplementary tables 1–3). The CERAD test results differed between participants with and without hypertension (*p* = 0.027 for the interaction), while other factors had no significant effect on cognitive function (all *p* > 0.05). The AFT test score also differed between participants stratified by heart failure (*p* = 0.025 for interaction), while again other factors had no significant effect (*p* > 0.05). The DSST results did not differ between subgroups stratified by these clinical and demographic factors.

### 3.3 Sensitivity Analyses

This study included only NHANES data from 2011 to 2012 and 2013 to 2014, as only these two survey periods included three cognitive tests. To further test robustness, we conducted a sensitivity analysis in which regression was repeated after excluding individuals over 65 years old, as cognitive abilities may decline with age and bias the results. After fully adjusting for confounding factors, analysis revealed a significant negative correlation between AIP and cognitive function related to AFT (β = −7.20, 95% CI: −12.50, −1.90, *p* < 0.01). In addition, AIP values in the fourth quartile were negatively correlated with AFT score (β = −2.94, 95% CI: −5.56, −0.32, *p* < 0.05). Details are provided in table 3.

**TABLE 3:** Sensitivity analyses to assess the association of AIP with cognitive function.

	CERAD		AFT		DSST	
	Model 1	Model 2	Model 1	Model 2	Model 1	Model 2
AIP index (continuous)	−1.89 (−3.71, −0.07) *	−6.09 (−12.45, 0.27)	−0.32 (−1.89, 1.25)	−7.20 (−12.50, −1.90) **	−1.90 (−6.61, 2.80)	−11.47 (−26.33, 3.40)
AIP index (quartiles)						
Q1	Reference		Reference		Reference	
Q2	−1.06 (−2.70, 0.59)	−0.56 (−2.38, 1.25)	−1.16 (−2.57, 0.25)	−1.33 (−2.84, 0.18)	−1.05 (−5.26, 3.16)	0.39 (−3.83, 4.60)
Q3	−0.86 (−2.53, 0.80)	−0.19 (−2.39, 2.02)	−0.82 (−2.25, 0.62)	−1.57 (−3.41, 0.26)	−5.45 (−9.73, −1.17) *	−4.59 (−9.72, 0.53)
Q4	−1.42 (−3.06, 0.21)	−0.47 (−3.62, 2.68)	−0.75 (−2.16, 0.65)	−2.94 (−5.56, −0.32) *	−2.05 (−6.24, 2.14)	−2.77 (−10.08, 4.54)

Abbreviations: AFT: animal fluency test; DSST: digit symbol substitution test. Data are presented as β (95% confidence intervals). * P<0.05. ** P<0.01. Crude model adjusted for None; Model 1 adjusted for age and gender; Model 2 adjusted for gender, age, race, education, marital status, PIR, hypertension, heart failure, coronary heart disease, stroke, smoking, diabetes, drink, TG, HDL, BMI; CERAD: The Consortium to Establish a Registry for Alzheimer's Disease.

### 3.4 Non‐Linear Relationship Between AIP and Cognitive Function

**FIGURE 2:**
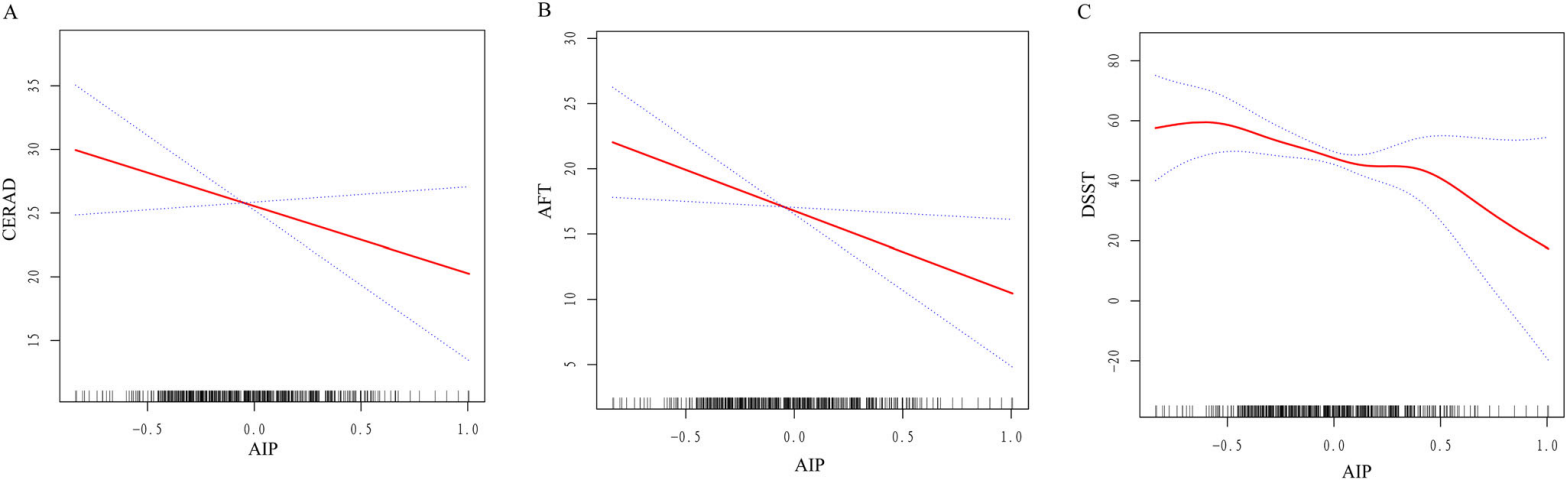
The association between AIP and cognitive performance related to (A) CERAD, (B) AFT, and (C) DSST.

Model 2 was constructed to assess nonlinear relationships between AIP and DSST (Figure 2). A piecewise regression model was used to set each interval to calculate the threshold effects (Supplementary table 4). For the relationship between AIP and cognitive function linked to DSST, we found an inflection point around 0.43. When AIP was less than 0.43, there was a moderate negative correlation (β = −26.25, 95% CI: −39.00, −13.50, *p* < 0.001), while when the AIP value was higher than 0.43, the negative correlation between AIP and DSST score was stronger (β = −66.47, 95% CI: −103.15, −29.78, *p* < 0.001).

## 4 Discussion

This study examined the association between AIP and three cognitive function tests among American civilians. In this cross‐sectional study that recruited 955 participants, we found that the fourth quartile of participants' AIP was significantly negatively associated with the AFT test, and the third quartile of participants' AIP was significantly negatively associated with the DSST test, after fully adjusting for confounding factors that could affect cognitive function. Based on the results of subgroup analysis, sensitivity analysis, and threshold analysis, we concluded that AIP is associated with AFT and DSST in cognitive function (table 4).

**TABLE 4:** Summary table of positive results for the association between AIP and cognitive function.

	AIP and CERAD		AIP and AFT		AIP and DSST	
	AIP index (continuous)	AIP index (quartiles)	AIP index (continuous)	AIP index (quartiles)	AIP index (continuous)	AIP index (quartiles)
Cognitive function β(95%CI)	none	none	−5.54(−9.26, −1.82)	Q3: ‐1.34(‐2.57, ‐0.10) Q4: ‐2.57(‐4.29, ‐0.86)	−15.79 (−26.32, −5.27)	Q3: ‐3.75 (‐7.26, ‐0.25)
Sensitivity analyses (age > 65 years old) β (95%CI)	none	none	−7.20 (−12.50, −1.90)	Q4: ‐2.94 (‐5.56, ‐0.32)	none	none

Abbreviations: AFT: Animal Fluency test; AIP: atherogenic index of plasma; DSST: Digit Symbol Substitution Test; CERAD: The Consortium to Establish a Registry for Alzheimer's Disease.

To the best of our knowledge, this is the first investigation on the association between AIP, a metric for atherosclerosis risk, and cognitive performance. The AIP is an accessible marker that has been associated with a number of vascular disorders. For instance, higher long‐term cumulative AIP and faster time course of cumulated AIP both increase the risk of myocardial infarction (Yuan et al. 2024), and current AIP has been associated with the risk of new‐onset hypertension (Zhang et al. 2023). Furthermore, a few studies have reported a strong correlation between higher AIP and imaging signs of intracranial and extracranial cerebrovascular disease with stenosis risk (Yu et al. 2023). The concomitant alterations in cerebral perfusion may in turn have deleterious effects on various neurocognitive processes. Indeed, a retrospective study of 334 participants reported that higher AIP was associated with more rapid neurological impairment following acute ischemic stroke (Q. Wang et al. 2023). Collectively, these findings suggest that the efficient management of AIP may delay the onset of cognitive impairment from cardiovascular and metabolic diseases. Furthermore, implementing early screening for elevated AIP levels could serve as an effective strategy for identifying individuals at heightened risk of future cognitive decline.

Cerebral ischemia and ensuing neuronal damage are frequent causes of cognitive dysfunction. Neurons have high metabolic rates but minimal energy reserves, resulting in susceptibility to ischemic and hypoxic damage; in fact, complete ischemia can lead to neuronal death after only a few minutes (Mehta et al. 2007). Some chronic systemic diseases, such as hypertension and diabetes, can also impair cognitive function by reducing the regional cerebral blood supply, and the incidences of hypertension and diabetes are closely related to higher AIP (Tan et al. 2023; Yin et al. 2023). In addition, cerebral ischemic damage is aggravated by the ensuing neuroinflammatory response driven by proinflammatory cytokines (Li et al. 2017). Thus, it is not surprising that higher AIP is predictive of cognitive impairment.

However, while high AIP was significantly associated with cognitive impairment as assessed by the AFT and DSST, there was no association with word recall as assessed by the CERAD. While bias from unadjusted covariates is possible, these results suggest that the effects of high AIP on cognition may differ across domains. The AFT assesses language fluency and executive function, while the DSST gauges processing speed, suggesting that a high AIP (and associated dyslipidemia) has a greater impact on executive and information processing than on short‐term verbal memory. Parthasarathy et al. (2017) reported a negative correlation between triglyceride levels and executive function in normal elderly individuals, and this association remained significant even after adjustment for various potential confounding variables. Similarly, a population‐based cohort study found that hypercholesterolemia (high total cholesterol, low HDL, and high LDL levels) in elderly males increased the risk of poor executive function by 25% and language fluency by 50% (Ancelin et al. 2014). Alternatively, the CERAD test focuses on delayed recall ability, and the association between AIP and CERAD was significant only after adjusting for age and sex, which may be related to the sample size of this study. Due to the limited inclusion of data on cognitive abilities in the NHANES database for the years 2011–2014, this study may not be able to detect differences between the AIP and CERAD tests after adjusting for variables.

The relationship of AIP with DSST was nonlinear, suggesting multifactorial mediation. Thus, the associations may be highly sensitive to threshold effects, statistical methods, and study design. A threshold effect was detected between the AIP and DSST, suggesting a phased relationship. However, as NHANES data were collected through complex sampling, we added weight adjustments during the statistical analysis. In addition, the selected covariates were closely related to AIP and cognitive function, ensuring the sensitivity and adaptability of the data collection method to capture potential nonlinear changes. Therefore, this association is complex but nonetheless robust.

The mechanisms mediating these associations of AIP with cognitive impairment as assessed by the AFT and DSST are still unclear, but may be related to cerebral atherosclerosis and neuroinflammation. First, AIP is reflective of excess cholesterol removal (cholesterol reverse transport). Therefore, a higher AIP may indicate that adipocytes are converting excess triglycerides (TG) into fat for storage, a process that can lead to the accumulation of cholesterol crystals in the intima of atherosclerotic vessels, luminal stenosis, and blockage (Huang et al. 2023). Atherosclerosis also increases the risk of various chronic metabolic diseases, including hypertension and diabetes, both of which are important risk factors for the impairment of executive function and information processing ability measured by the AFT and DSST (Moheet, Mangia, and Seaquist 2015; Santisteban et al. 2023). In addition, AIP is considered a substitute indicator for low‐density LDL (sdLDL) and is negatively correlated with the size of LDL cholesterol particles. A higher AIP thus indicates a decrease in LDL particle diameter and an increase in the proportion of sdLDL particles. These small sdLDL particles are cleared slowly from the blood and so are more likely to be converted into oxidized LDL, which can lead to inflammatory reactions and the development of atherosclerosis (Dobiásová and Frohlich 2001). Related studies have shown that an increase in inflammation levels can reduce cognitive abilities related to AFT and DSST performance (Guo et al. 2024; Sun et al. 2022).

This study incorporated several design attributes advantageous for establishing a relationship between AIP and cognitive function. First, we used population‐based survey data gathered using preset methods to obtain a nationally representative sample. Second, subgroup analyses were used to confirm the stability of the results and account for differences in age, potentially the strongest confound variable. Similarly, multiple models with adjustments for various potential covariates were constructed and compared, including a model for a nonlinear association that may be missed by linear regression analysis. The large sample size and adjustment for many potential confounding variables undoubtedly enhanced the scientific validity and precision of the study. However, the current study has several limitations. First, the cross‐section design cannot establish a causal relationship. It is thus uncertain if AIP directly influences cognitive outcomes. To enhance our understanding of this relationship, future research should focus on larger, longitudinal studies that can track changes in AIP alongside cognitive performance over time. Additionally, the cognitive function data analyzed in this study were confined to the 2011–2014 NHANES cycles, which may hinder the applicability of our findings to various populations or over different time periods. Given that cognitive function is influenced by numerous factors such as aging, socio‐economic status, and lifestyle changes, more extensive cohort studies with the potential for subgroup comparisons are necessary to confirm our findings and strengthen the validity of our conclusions. Moreover, atrial fibrillation and cerebrovascular atherosclerotic diseases (such as carotid or vertebral artery stenosis) are well‐established risk factors for cognitive impairment and may represent potential confounders in this study. However, due to the lack of systematic collection of diagnostic information on atrial fibrillation or carotid artery stenosis during the study period in the dataset utilized, we were unable to include these variables as covariates in the multivariable regression models. This limitation may have an impact on the conclusions drawn from this study. In addition, several potential confounding variables that may impact both AIP and cognitive performance were not accounted for in this analysis, including physical activity, diet, mental health status, and medication use. The absence of these covariates may lead to residual confounds, obscuring the true nature of the relationship between AIP and cognitive performance. Furthermore, due to the presence of missing data, this study may still be subject to potential bias arising from loss to follow‐up and sample selection. Therefore, future studies should include a wider array of potential confounders and more complete data for a more nuanced understanding of these dynamics.

## 5 Conclusions

In summary, these findings indicated a correlation between the AIP and cognitive function as assessed by the AFT and DSST. Further research is necessary to investigate the causal links between high AIP and cognitive dysfunction.

## Author Contributions

**Pingping Huang**: conceptualization, investigation, software, formal analysis, data curation, writing – review and editing, writing – original draft, visualization. **Gaocan Ren**: conceptualization, investigation, writing – review and editing. **Yifei Wang**: conceptualization, writing – review and editing. **Yicheng Liu**: writing – review and editing, conceptualization. **Hongwei Zhang**: writing – review and editing, visualization. **Shuangqing Fu**: conceptualization. **Zhibo Zhang**: conceptualization. **Lijun Guo**: supervision. **Xiaochang Ma**: supervision.

## Ethics Statement

The National Centre for Health Statistics' Research Ethics Review Board approved the study in order to guarantee ethical standards. Every participant in the study gave informed consent before to participation.

## Conflicts of Interest

The authors declare that the research was conducted in the absence of any commercial or financial relationship that could be construed as a potential conflict of interest.

## Peer Review

The peer review history for this article is available at https://publons.com/publon/10.1002/brb3.70768

## Supporting information




**Supplementary Tables**: brb370768‐sup‐0001‐SuppMat.docx

## Data Availability

The original contributions presented in the study are included in the article/supplementary material. Further inquiries can be directed to the corresponding authors.
