# Supplementary material for: The Association Between the Atherogenic Index of Plasma and Cognitive Function: Evidence From the NHANES 2011–2014
Source: Brain Behav. 2025 Aug 22;15(8):e70768. doi: 10.1002/brb3.70768 (PMC12373517; doi:10.1002/brb3.70768)
Supplement: Supplementary file 1 — Supplementary Tables: brb370768‐sup‐0001‐SuppMat.docx [file BRB3-15-e70768-s001.docx]

Supplementary materials

Table 1Stratified analysis of the relationship between AIP and cognitive function based on CERAD

| Subgroup | **β** | *P*-value | *P* for interaction |
| --- | --- | --- | --- |
| Gender |  |  | 0.174 |
| male | -2.13 (-7.61, 3.36) | 0.448 |  |
| female | -4.89 (-9.66, -0.11) | 0.045 |  |
| Age |  |  | 0.180 |
| ＜70 | -5.61 (-11.17, -0.06) | 0.048 |  |
| ≥70 | 0.90 (-7.16, 8.95) | 0.827 |  |
| BMI |  |  | 0.282 |
| <25 | -10.56 (-19.81, -1.32) | 0.025 |  |
| 25-29.9 | -5.03 (-12.60, 2.54) | 0.192 |  |
| ≥30 | -0.32 (-9.27, 8.64) | 0.945 |  |
| PRI |  |  | 0.991 |
| ≤1 | -4.32 (-10.56, 1.93) | 0.175 |  |
| ＞1 | -4.29 (-8.99, 0.41) | 0.074 |  |
| Drinking |  |  | 0.280 |
| yes | -2.35 (-7.74, 3.03) | 0.392 |  |
| no | -8.01 (-17.02, 0.99) | 0.081 |  |
| Smoking |  |  | 0.204 |
| yes | -3.26 (-8.14, 1.61) | 0.189 |  |
| no | -5.72 (-10.92, -0.53) | 0.031 |  |
| Hypertension |  |  | 0.027 |
| yes | -0.01 (-5.92, 5.90) | 0.996 |  |
| no | -10.46 (-17.80, -3.12) | 0.005 |  |
| Heart failure |  |  | 0.182 |
| yes | 9.80 (-11.51, 31.10) | 0.367 |  |
| no | -4.74 (-9.48, 0.01) | 0.051 |  |
| CHD |  |  | 0.187 |
| yes | 9.43 (-11.85, 30.71) | 0.385 |  |
| no | -4.87 (-9.62, -0.11) | 0.045 |  |
| Stroke |  |  | 0.668 |
| yes | -7.92 (-26.44, 10.59) | 0.401 |  |
| no | -3.81 (-8.60, 0.97) | 0.118 |  |
| Diabetes |  |  | 0.33 |
| yes | -2.13 (-12.31, 8.06) | 0.682 |  |
| no | -6.07 (-11.62, -0.52) | 0.032 |  |
| Borderline | 10.06 (-12.92, 33.05) | 0.391 |  |

Abbreviations: Ratio of family income to poverty (PRI); coronary heart disease (CHD)

Table 2 Stratified analysis of the relationship between AIP and cognitive function based on AFT

| Subgroup | **β** | *P*-value | *P* for interaction |
| --- | --- | --- | --- |
| Gender |  |  | 0.855 |
| male | -2.99 (-8.08, 2.10) | 0.250 |  |
| female | -3.70 (-9.63, 2.24) | 0.222 |  |
| Age |  |  | 0.450 |
| ＜70 | -5.15 (-9.63, -0.67) | 0.024 |  |
| ≥70 | -2.19 (-8.65, 4.27) | 0.506 |  |
| BMI |  |  | 0.734 |
| <25 | -3.84 (-11.28, 3.61) | 0.312 |  |
| 25-29.9 | -6.46 (-12.58, -0.35) | 0.038 |  |
| ≥30 | -3.02 (-10.22, 4.18) | 0.411 |  |
| PRI |  |  | 0.282 |
| ≤1 | -0.39 (-9.27, 8.49) | 0.931 |  |
| ＞1 | -5.67 (-9.78, -1.57) | 0.006 |  |
| Drinking |  |  | 0.466 |
| yes | -4.12 (-8.09, -0.15) | 0.042 |  |
| no | -5.85 (-10.94, -0.77) | 0.024 |  |
| Smoking |  |  | 0.316 |
| yes | -3.62 (-7.77, 0.53) | 0.087 |  |
| no | -5.73 (-10.16, -1.30) | 0.011 |  |
| Hypertension |  |  | 0.153 |
| yes | -3.48 (-7.53, 0.57) | 0.092 |  |
| no | -6.52 (-11.07, -1.96) | 0.005 |  |
| Heart failure |  |  | 0.025 |
| yes | 15.01 (-2.82, 32.84) | 0.099 |  |
| no | -5.19 (-9.01, -1.37) | 0.007 |  |
| Coronary heart disease |  |  | 0.090 |
| yes | 8.36 (-7.33, 24.04) | 0.296 |  |
| no | -5.32 (-9.16, -1.47) | 0.006 |  |
| Stroke |  |  | 0.728 |
| yes | -6.11 (-15.26, 3.04) | 0.191 |  |
| no | -4.56 (-8.32, -0.79) | 0.017 |  |
| Diabetes |  |  | 0.757 |
| yes | -2.42 (-10.36, 5.52) | 0.550 |  |
| no | -5.71 (-10.18, -1.24) | 0.012 |  |
| Borderline | -3.28 (-20.76, 14.20) | 0.713 |  |

Abbreviations: Ratio of family income to poverty (PRI);coronary heart disease(CHD)

Table 3 Stratified analysis of the relationship between AIP and cognitive function based on DSST

| Subgroup | **β** | *P*-value | *P* for interaction |
| --- | --- | --- | --- |
| Gender |  |  | 0.160 |
| male | -6.81 (-19.55, 5.94) | 0.295 |  |
| female | -13.43 (-24.52, -2.34) | 0.017 |  |
| Age |  |  | 0.716 |
| ＜70 | -10.81 (-23.53, 1.91) | 0.096 |  |
| ≥70 | -6.77 (-25.19, 11.66) | 0.471 |  |
| BMI |  |  | 0.455 |
| <25 | -3.76 (-25.28, 17.77) | 0.732 |  |
| 25-29.9 | -11.91 (-29.49, 5.68) | 0.185 |  |
| ≥30 | -22.45 (-43.32, -1.58) | 0.035 |  |
| PRI |  |  | 0.440 |
| ≤1 | -3.44 (-29.63, 22.76) | 0.797 |  |
| ＞1 | -14.59 (-26.52, -2.66) | 0.016 |  |
| Drinking |  |  | 0.943 |
| yes | -13.51 (-24.84, -2.19) | 0.019 |  |
| no | -13.15 (-25.77, -0.52) | 0.041 |  |
| Smoking |  |  | 0.604 |
| yes | -11.21 (-22.55, 0.13) | 0.052 |  |
| no | -13.55 (-25.64, -1.45) | 0.028 |  |
| Hypertension |  |  | 0.299 |
| yes | -10.72 (-22.04, 0.59) | 0.063 |  |
| no | -15.57 (-27.83, -3.32) | 0.012 |  |
| Heart failure |  |  | 0.885 |
| yes | -14.23 (-43.09, 14.62) | 0.333 |  |
| no | -12.22 (-23.09, -1.36) | 0.027 |  |
| Coronary heart disease |  |  | 0.939 |
| yes | -12.21 (-26.36, 1.94) | 0.091 |  |
| no | -12.60 (-23.42, -1.79) | 0.022 |  |
| Stroke |  |  | 0.944 |
| yes | -11.50 (-38.06, 15.07) | 0.396 |  |
| no | -12.40 (-23.28, -1.52) | 0.025 |  |
| Diabetes |  |  | 0.689 |
| yes | -7.11 (-29.87, 15.66) | 0.540 |  |
| no | -16.42 (-29.28, -3.56) | 0.012 |  |
| Borderline | -25.94 (-73.87, 22.00) | 0.289 |  |

Abbreviations: Ratio of family income to poverty (PRI);coronary heart disease(CHD)

Table 4 Threshold effect analysis for correlations between the AIP and cognitive performance

| Outcomes | DSST |
| --- | --- |
| Linear effect model |  |
| **β**, (95% CI), *p* value | -15.79 (-26.32, -5.27) 0.0033 |
| Non-linear model |  |
| Infection point (K) | 0.43 |
| **β**1, (95% CI)(<K), *p* value | -26.25 (-39.00, -13.50) <0.001 |
| **β**2, (95% CI)(>K), *p* value | -66.47 (-103.15, -29.78) <0.001 |
| LLR | 0.004 |

Abbreviation: logarithmic likelihood ratio test *p* value (LLR)
